# Inhibition of NAPDH Oxidase 2 (NOX2) Prevents Oxidative Stress and Mitochondrial Abnormalities Caused by Saturated Fat in Cardiomyocytes

**DOI:** 10.1371/journal.pone.0145750

**Published:** 2016-01-12

**Authors:** Leroy C. Joseph, Emanuele Barca, Prakash Subramanyam, Michael Komrowski, Utpal Pajvani, Henry M. Colecraft, Michio Hirano, John P. Morrow

**Affiliations:** 1 Department of Medicine, College of Physicians and Surgeons of Columbia University, New York, New York, United States of America; 2 Department of Neurology, College of Physicians and Surgeons of Columbia University, New York, New York, United States of America; 3 Department of Physiology and Cellular Biophysics, Columbia University, New York, New York, United States of America; Stellenbosch University, SOUTH AFRICA

## Abstract

Obesity and high saturated fat intake increase the risk of heart failure and arrhythmias. The molecular mechanisms are poorly understood. We hypothesized that physiologic levels of saturated fat could increase mitochondrial reactive oxygen species (ROS) in cardiomyocytes, leading to abnormalities of calcium homeostasis and mitochondrial function. We investigated the effect of saturated fat on mitochondrial function and calcium homeostasis in isolated ventricular myocytes. The saturated fatty acid palmitate causes a decrease in mitochondrial respiration in cardiomyocytes. Palmitate, but not the monounsaturated fatty acid oleate, causes an increase in both total cellular ROS and mitochondrial ROS. Palmitate depolarizes the mitochondrial inner membrane and causes mitochondrial calcium overload by increasing sarcoplasmic reticulum calcium leak. Inhibitors of PKC or NOX2 prevent mitochondrial dysfunction and the increase in ROS, demonstrating that PKC-NOX2 activation is also required for amplification of palmitate induced-ROS. Cardiomyocytes from mice with genetic deletion of NOX2 do not have palmitate-induced ROS or mitochondrial dysfunction. We conclude that palmitate induces mitochondrial ROS that is amplified by NOX2, causing greater mitochondrial ROS generation and partial depolarization of the mitochondrial inner membrane. The abnormal sarcoplasmic reticulum calcium leak caused by palmitate could promote arrhythmia and heart failure. NOX2 inhibition is a potential therapy for heart disease caused by diabetes or obesity.

## Introduction

Excessive lipid accumulation is found in cardiomyocytes from obese and diabetic patients, and is believed to contribute to heart failure and arrhythmia [1–4]. Obesity and diabetes increase the risk of heart failure, independently of coronary atherosclerosis [5–7]. Obese and diabetic patients are at increased risk for several types of arrhythmia, including atrial fibrillation [8, 9]. More importantly, several epidemiologic studies have shown that obese patients have approximately twice the risk of sudden cardiac death, and diabetics three times the risk, as age matched controls [10–13]. The increased risk of sudden cardiac death is greater than the increased risk of myocardial infarction, suggesting that arrhythmic events are increased more than coronary events in obese and diabetic patients. Human epidemiology studies show that higher saturated fat intake leads to an increased risk of sudden cardiac death, [14–17], suggesting that the effects of saturated fat on the heart may be more important than obesity per se.

Reactive oxygen species (ROS) are a mechanistic link between lipid metabolism and cardiovascular pathology [18–20]. Mild, transient increases in cardiac ROS may be involved in adaptive processes, but it is postulated that long-term increases in cardiac ROS are detrimental [21]. There are several sources of ROS in cardiomyocytes, including NAPDH oxidase (NOX), nitric oxide synthase (NOS), and mitochondria. Mitochondria are a major source of ROS in myocytes [22]. A high-fat diet increases mitochondrial ROS in skeletal muscle [23] and decreases cardiac efficiency, defined as cardiac work divided by oxygen consumption [24]. However, the molecular mechanisms are not well understood, despite the fact that isolated mitochondria have been studied intensely for decades. Experiments using isolated cardiac mitochondria exposed to saturated fatty acids have given conflicting results regarding ROS generation [25, 26]. There is relatively little data regarding the effects of fatty acid metabolism on ROS and mitochondrial function in intact cardiomyocytes. Using intact cardiomyocytes has the advantage of preserving signaling pathways and interactions between mitochondria and other subcellular compartments.

In order to study the effects of fatty acids on cardiac metabolism, we used palmitate, because it is one of the most prevalent saturated fats in the bloodstream of mammals [27]. We used the monounsaturated fatty acid oleate (the principle component of olive oil) as a control, which is thought to be benign based on nutritional epidemiology [28]. We hypothesized that physiologic levels of saturated fatty acid could increase mitochondrial ROS in cardiomyocytes, leading to abnormalities of calcium homeostasis and mitochondrial function.

## Materials and Methods

### Materials

Oleate and palmitate were purchased from Sigma and dissolved in sterile water to make a 10 mM solution with 10% fatty-acid free BSA (Sigma), then diluted to final concentration 200 μM in media. Mito-TEMPO was purchased from Enzo Life Science. The NOX2 inhibitor peptide gp91-ds tat was purchased from Anaspec, Inc. Mitosox red, Rhod2-AM, and TMRM were purchased from Life Technologies. Other chemicals were purchased from Sigma. The anti-PKCalpha antibody was purchased from Santa Cruz. The anti-KDEL primary antibody was purchased from Thermo Scientific.

### Animal care and cardiomyocyte isolation

Animal protocols were approved by the Columbia University Institutional Animal Care and Use Committee and were carried out in accordance with the NIH guidelines for the care and use of laboratory animals. Wild type (WT) C57BL and B6.129S-*Cybb*^*tm1Din*^/J (NOX2 KO) mice were purchased from Jackson labs. Mice were 9–12 weeks old at the time of experiments. Isolation of cardiomyocytes was performed as previously described using isoflurane for anesthesia [2].

### Fatty acid treatment and measurements of ROS, mitochondrial calcium, and mitochondria depolarization

We used a physiologic concentrations of fatty acids, (200 μM) bound to bovine serum albumin (BSA) [29]. After fatty acid treatment, isolated cardiomyocytes were divided into aliquots. To measure total cellular ROS, cells were loaded with the fluorescent dye 2’, 7’-Dichlorofluorescin diacetate (H2DCF-DA) (25 μM). Once it enters the cell, H2DCF-DA is converted by oxidation to DCF, which is fluorescent, and this is widely used as a measure of general oxidative stress [30]. To measure mitochondrial ROS, Mitosox Red (5 μM), was used to analyze mitochondrial superoxide generation. Cells were incubated with DCF or mitosox red for 30 minutes in the dark. Excess DCF or Mitosox Red was removed with two washes of BSA solution. DCF fluorescence was recorded at excitation/emission wavelengths: 488/532 nm whereas MitoSOX Red was recorded at 525 (excitation) and 620 nm (emission). To assess the changes in mitochondrial membrane potential, cardiomyocytes were stained with 1nM tetramethylrhodamine methyl (TMRM) ester for 30 minutes and recorded at 543 nm (excitation)/590 nm (emission). TMRM preferentially accumulates in mitochondria due to its positive charge. As mitochondria are depolarized, they trap less TMRM, and so the signal is proportional to the inner membrane potential of the mitochondria. To determine the changes in mitochondrial calcium in living cells, cardiomyocytes were loaded with 10 μM Rhod 2-AM and incubated for 30 minutes followed by a one-hour washout with cytosolic quenching using manganese, to produce specific mitochondrial calcium signal [31]. Rhod2AM fluorescence was measured at 552 nm (excitation)/ 581 nm (emission). Cells were loaded onto a 96-well in triplicate, plated at 2000 cells/ well, and fluorescence was measured with a Tecan Infinite 200 plate reader. NADPH-dependent superoxide production was measured in LV homogenates using lucigenin enhanced chemiluminescence as previously described [32].

### Cell culture

H9c2 cells were purchased from ATCC and cultured in DMEM (Life Technologies) supplemented with 10% FBS (Atlanta Biologicals) and with penicillin and streptomycin (Fisher).

### Respirometry

Oxygen consumption rate and extra-cellular acidification rate were measured in cardiomyocytes with a XF24 Extracellular Flux Analyzer (Seahorse Bioscience, Billerica, MA, USA). Cardiomyocytes were seeded in XF 24-well cell culture microplates at a density of 3000 cells/well. The wells were pre-coated with laminin to improve adherence. Cells were then treated with 200 μM of BSA-conjugated fatty acid: palmitate, oleate or BSA vehicle control. After replacing the growth medium with 525 μL of bicarbonate-free DMEM pre-warmed at 37°, cells were incubated for 30 min before starting the assay procedure. After baseline measurements, ATP synthase was inhibited with oligomycin, and then maximal respiration was stimulated with the ionophore carbonyl cyanide-p-trifluoromethoxyphenylhydrazone (FCCP). Finally, antimycin-A and rotenone were added to inhibit the mitochondrial electron transport chain in order to measure non-mitochondrial oxygen consumption. The concentrations used are as follows: oligomycin (4 μM), carbonyl cyanide 4-(trifluoromethoxy) phenylhydrazone (FCCP, 0.25 μM), and rotenone plus antimycin A (1 μM each). We determined the best concentration if FCCP for cardiomyocytes by conducting a series of titration experiments at the beginning of the project. Coupling efficiency (defined as the reduction in oxygen consumption after oligomycin injection) is the fraction of basal mitochondrial oxygen consumption used for ATP synthesis. Cell respiratory control ratio is the ratio of the uncoupled oxygen consumption rate to the oxygen consumption rate with oligomycin. Non-mitochondrial respiration was subtracted from all rates.

### Respiratory chain enzyme activity assessment

Biochemical activities of COX, NADH-cytochrome c reductase (complex I+III), succinate-cytochrome c reductase (complex II+III), NADH-CoQ reductase (complex I), succinate dehydrogenase (complex II) and citrate synthase were assayed with spectrophotometry as previously described [33]. CoQ-cytochrome c reductase (complex III) was measured as described previously [34]. Respiratory chain enzyme activity values were normalized to protein concentration.

### Preparation of protein extracts and western blot analysis

For total protein extract preparation, mouse ventricular samples or cell pellets were homogenized in RIPA buffer (Thermo Scientific) containing protease and phosphatase inhibitors (Roche). For membrane extract, ventricular samples were homogenized in membrane buffer (20 mM Tris-HCl pH 7.2, 2 mM EDTA, 2 mM EGTA, 50 mM NaF, and 6 mM mercaptoethanol) containing protease and phosphatase inhibitors (Roche). Samples were spun at 33, 000 rpm for 30 minutes at 2°C. The pellet was suspended in buffer containing 1% SDS and incubated on ice for 30 minutes. Samples were subjected to 10% SDS-PAGE. Proteins were transferred onto a nitrocellulose membrane and probed with the indicated antibodies. PKCalpha primary antibody was used at a 1:1000 dilution. The blots were reprobed with KDEL primary antibody for normalization. Alkaline phosphatase-conjugated goat anti-rabbit IgG (Santa Cruz) was used as secondary antibody, and immunoreactive bands were detected with ECL solution (Thermo Scientific), according to the manufacturer’s instructions. Quantification was performed using Image J software.

### Spark recording and analysis

Sparks were recorded with a Leica SP2 confocal microscope equipped with a 63X 1.4 NA objective. Cardiomyocytes were loaded with fluo-4 (5 mM, 10 min), in modified Tyrode’s solution containing 1 mM calcium. Line scan images were recorded with Leica TCS software and quantified with the Sparkmaster plugin of ImageJ, created by Donald Bers.

### Confocal microscopy

Confocal imaging was performed on a Nikon A1 laser scanning confocal attachment on an Eclipse Ti microscope stand (Nikon Instruments, Melville, NY) using a 40x/1.3 Plan Fluor oil-immersion objective. The confocal pinhole was set at 1 Airy unit, to produce an optical section of approximately 0.5 um. MitoTracker Green and MitoSOX Red (Life Technologies, Grand Island, NY) were imaged sequentially and emission was collected with standard fluorescein and rhodamine emission filters. Single optical sections were collected for each cell shown.

### Statistical analysis

Results are presented as mean ±SEM. The unpaired t-test was used for comparisons of two means; a 2-tailed value of *P* < 0.05 was considered statistically significant. For groups of 2 or more ANOVA was used with post-hoc testing (Prism v5, GraphPad Software).

## Results

### The saturated fat palmitate causes a decrease in mitochondrial respiration in cardiomyocytes

**Fig 1:**
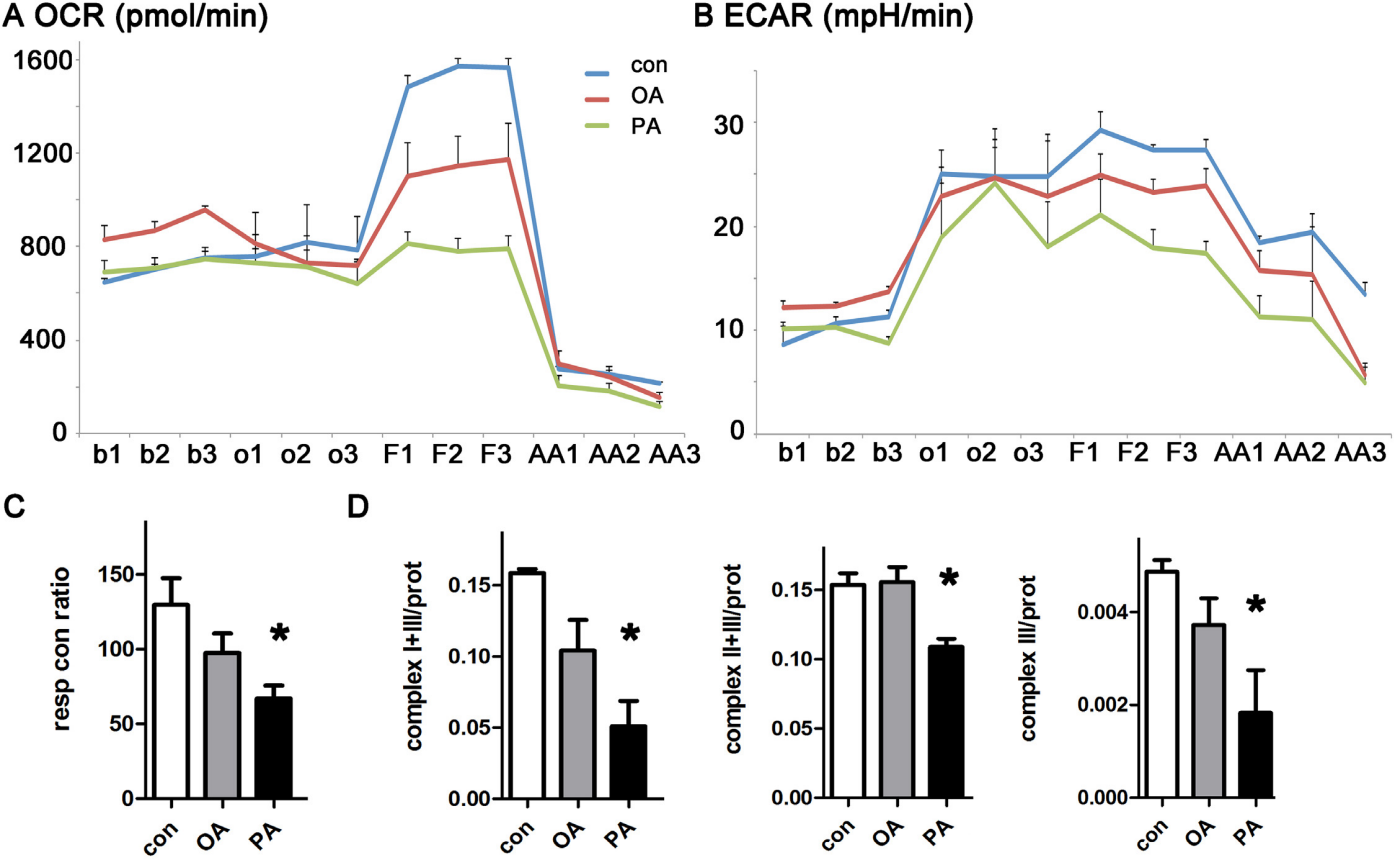
Palmitate causes a decrease in mitochondrial oxygen consumption in cardiomyocytes. A. Oxygen consumption rate is decreased by palmitate. B. Extracellular acidification rate from the same experiment. Data from WT cardiomyocytes. Measurements from three time-points were obtained under each condition, using triplicate wells. b = baseline, o = oligomycin, F = FCCP, AA = antimycin-A and rotenone. C. Respiratory control ratio is reduced significantly by palmitate. D. Electron transport chain complex III activity is significantly reduced by palmitate. Units are nanomoles substrate utilized/min/mg protein. The means of all graphs are significantly different by ANOVA, * = sig different from control by post-hoc test.

We measured the effect of fatty acids on mitochondrial respiration using isolated adult mouse ventricular myocytes from WT mice. We treated cardiomyocytes with palmitate, oleate, or BSA (vehicle control) for 3 hours before flux analysis. We quantified respiration by measuring oxygen consumption rate and estimated glycolysis by extracellular acidification rate. Palmitate had a strong inhibitory effect on maximal respiration (Fig 1A). Oleate had an intermediate effect. Palmitate also caused a small reduction in glycolysis, perhaps as compensation for using fatty acid as a fuel source (Fig 1B). Additional experiments were performed to evaluate mitochondrial enzyme activity, using lysates made from cardiomyocytes after exposure to oleate or palmitate. Palmitate caused a significant reduction in the activity of mitochondrial complex III of the electron transport chain (Fig 1D).

### Palmitate increases mitochondrial ROS in cardiomyocytes

**Fig 2:**
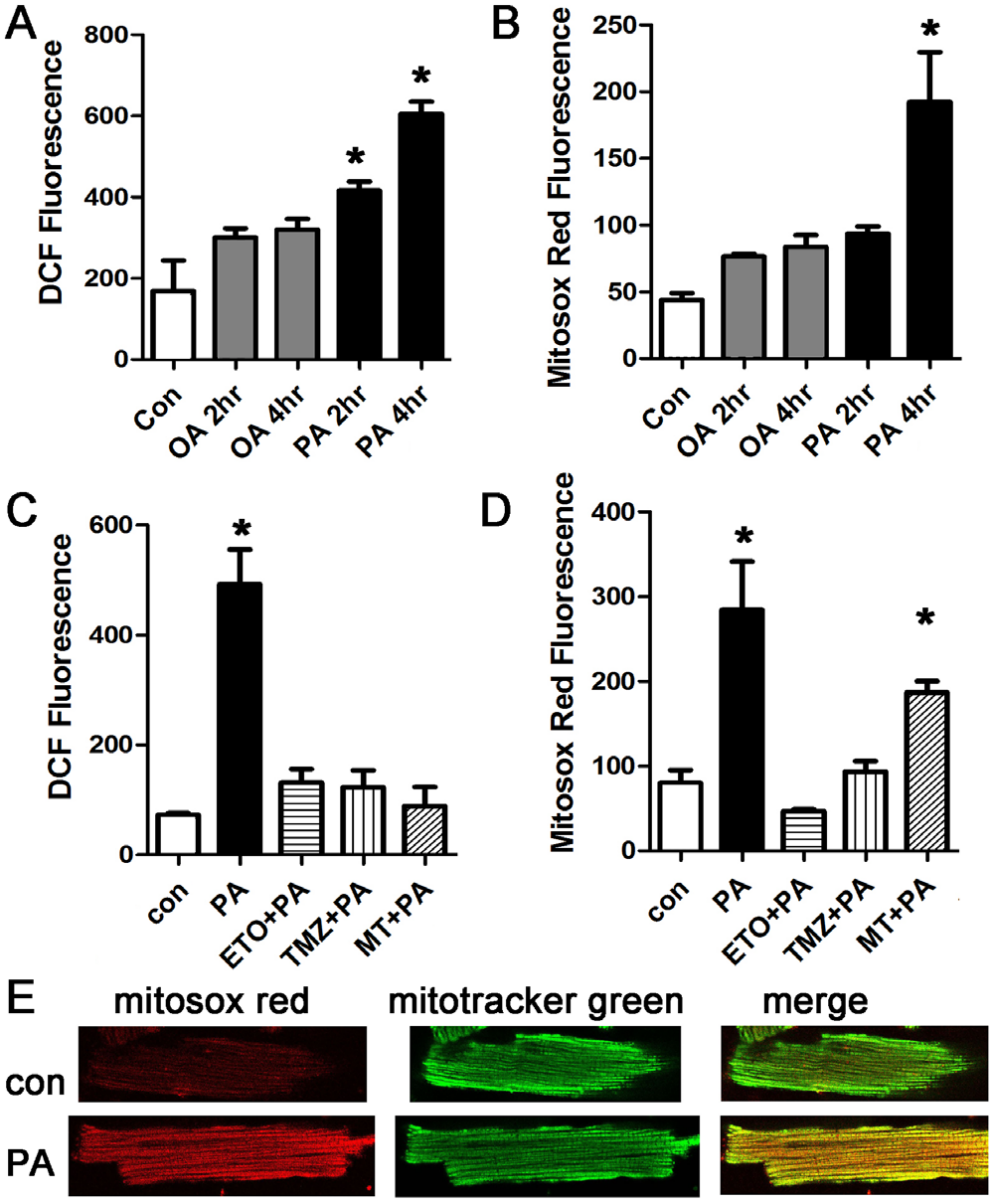
Cardiomyocytes treated with palmitate have increased total and mitochondrial ROS. A. Representative experiment done with cardiomyocytes in triplicate, height is DCF fluorescence minus background, in live cells, expressed in arbitrary units, mean + SEM. Palmitate, but not oleate, increases ROS significantly. PA = palmitate 200 μM, OA = oleate 200 μM, B. Cardiomyocytes from the same experiment using mitosox red readout to indicate mitochondrial ROS. C. Inhibition of mitochondrial lipid uptake or beta-oxidation prevents the increase in ROS. ETO = etomoxir 200 μM, TMZ = trimetazidine 10 μM, MT = mito-TEMPO, 20 μM. D. Cardiomyocytes from the same experiment using mitosox red readout. For all panels, means are significantly different by ANOVA, * = sig different from control by post-hoc test. E. Confocal images of live isolated cardiomyocytes after control and palmitate treatment. Cells were stained with mitosox red and counter-stained with mitotracker green.

Next, we quantified palmitate-induced ROS production in intact cardiomyocytes. Cardiomyocytes were treated with a physiologic dose of palmitate or oleate for two or four hours and then stained with the ROS indicators DCF and mitosox red (a specific indicator of mitochondrial ROS). At both time-points, palmitate significantly increased total cellular ROS, as indicated by DCF signal (Fig 2A). Palmitate also caused a significant increase in mitochondrial ROS (Fig 2B and 2E). Oleate did not cause a significant increase in total or mitochondrial ROS. The fact that total ROS is increased at an earlier time point than mitochondrial ROS suggests that non-mitochondrial sources of ROS have an important contribution to total ROS induced by saturated fat. These results show that a shorter exposure to saturated fat may be harmless, but longer durations of exposure increase ROS. Human studies have shown that high fat diet causes persistent elevations of serum fatty acids (longer than 4 hours) [35], supporting the physiologic relevance of this duration of treatment.

To determine if mitochondrial use of fatty acids as a metabolic substrate was required for the increase in ROS, we used a pharmacologic approach. Both etomoxir, an inhibitor of mitochondrial fatty-acid uptake, and trimetazidine, an inhibitor of fatty acid beta-oxidation, prevented the increase in total cellular and mitochondrial ROS (Fig 2C and 2D). In addition, mito-tempo, a mitochondrial targeted anti-oxidant, eliminated the increase in total ROS. Mito-tempo suppresses the increase in total ROS more effectively than it suppresses the increase in mitochondrial ROS. This suggests that the large increase in total cellular ROS at the later time-point may be amplified by a threshold level of mitochondrial ROS.

To verify these findings, and to demonstrate that the palmitate effect is not a consequence of the isolation procedure, we repeated these experiments using the cardiomyoblast cell line H9c2. These experiments also showed increased ROS after exposure to palmitate (S1 file). Since high doses of palmitate can cause apoptosis in some cell types, cell death was evaluated by trypan blue. At these doses and time points, palmitate did not cause cell death. There was a non-significant trend towards increased cell death with concentrations of palmitate greater than 200 μM (S1 file).

### Palmitate depolarizes the mitochondrial inner membrane and causes mitochondrial calcium overload

**Fig 3:**
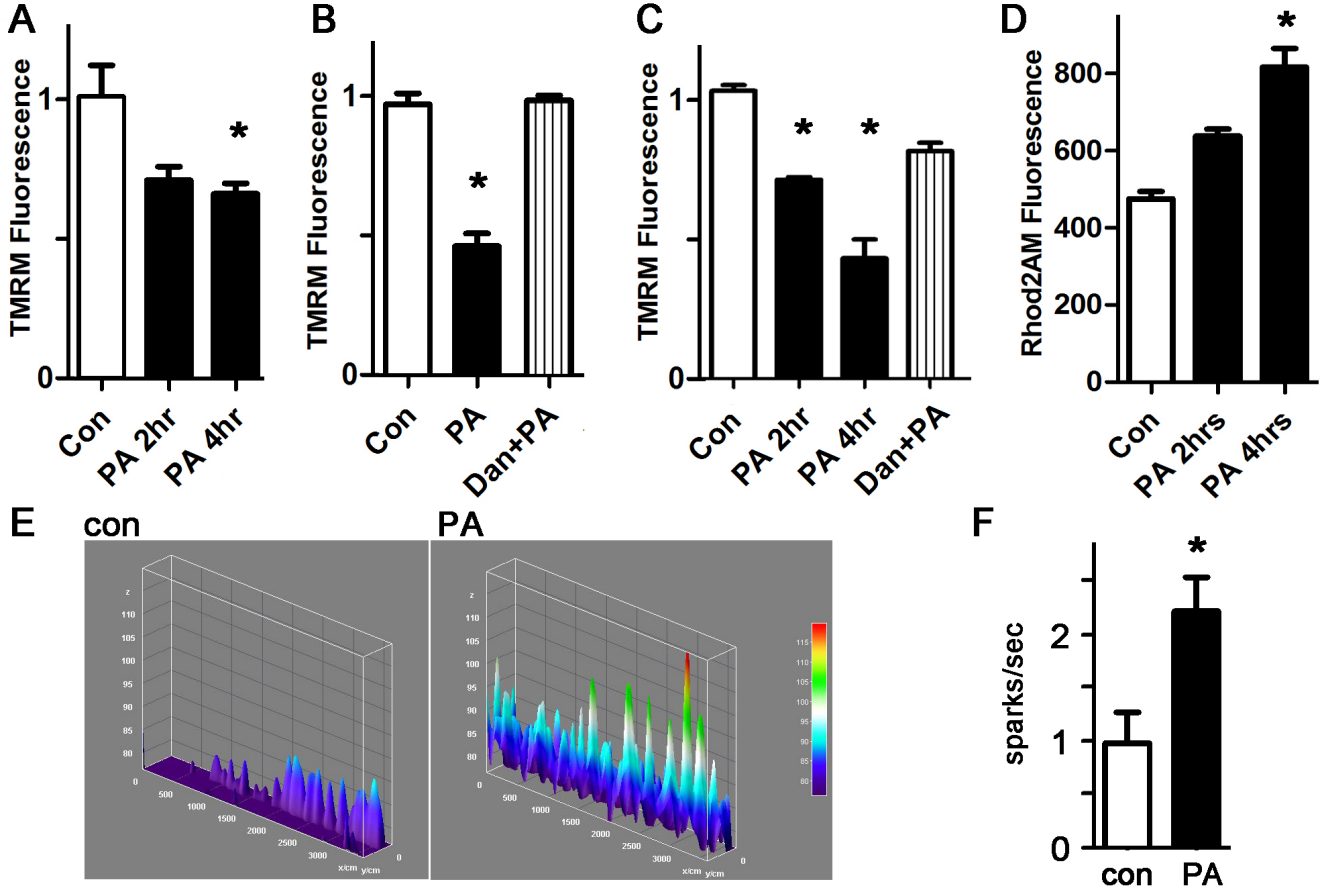
Palmitate depolarizes the mitochondrial inner membrane potential and causes mitochondrial calcium overload from increased sparks. A. Representative experiment done with cardiomyocytes in triplicate, height is TMRM fluorescence minus background, in live cells, expressed in arbitrary units, mean + SEM. PA = palmitate 200 μM. B. Dantrolene prevents the loss of inner membrane potential. Dan = dantrolene 1 μM. C. Similar experiment using H9c2 cells and TMRM readout. D. Cardiomyocytes with Rhod2AM readout indicating mitochondrial calcium. For all panels, means are significantly different by ANOVA, * = sig different from control by post-hoc test. E. Representative line-scan sparks experiments from cardiomyocytes; height is spark amplitude, over time. F. Graph of sparks, normalized to control, n = 10–11, * = p<0.05.

To better characterize the mitochondrial abnormalities induced by palmitate, we quantified the mitochondrial inner membrane potential using TMRM [36]. Palmitate causes a significant reduction in mitochondrial membrane potential in cardiomyocytes and H9c2 cells, indicating mitochondrial damage or dysfunction (Fig 3A and 3C). Dantrolene, a specific inhibitor of ryanodine receptor channels (RyR), prevented the decrease in mitochondrial membrane potential. Since RyR channels release calcium from the sarcoplasmic reticulum, this indicates that palmitate-induced ROS leads to increased calcium release from RyR channels, which enhances mitochondrial dysfunction. We verified this mechanism by quantifying mitochondrial calcium levels during palmitate treatment, which demonstrated that palmitate causes a significant increase in mitochondrial calcium (Fig 3D).

To confirm that mitochondrial calcium overload occurs by RyR-mediated calcium release from the sarcoplasmic reticulum, we quantified spontaneous calcium release events from the sarcoplasmic reticulum, in the form of sparks. Treatment of WT cardiomyocytes with palmitate caused a significant increase in spark frequency (Fig 3E and 3F). Our data show that palmitate exposure is sufficient to increase calcium leak from the sarcoplasmic reticulum, causing mitochondrial calcium overload in cardiomyocytes, a novel finding.

### Palmitate induced ROS production is inhibited by blocking protein kinase C or NAPDH oxidase

**Fig 4:**
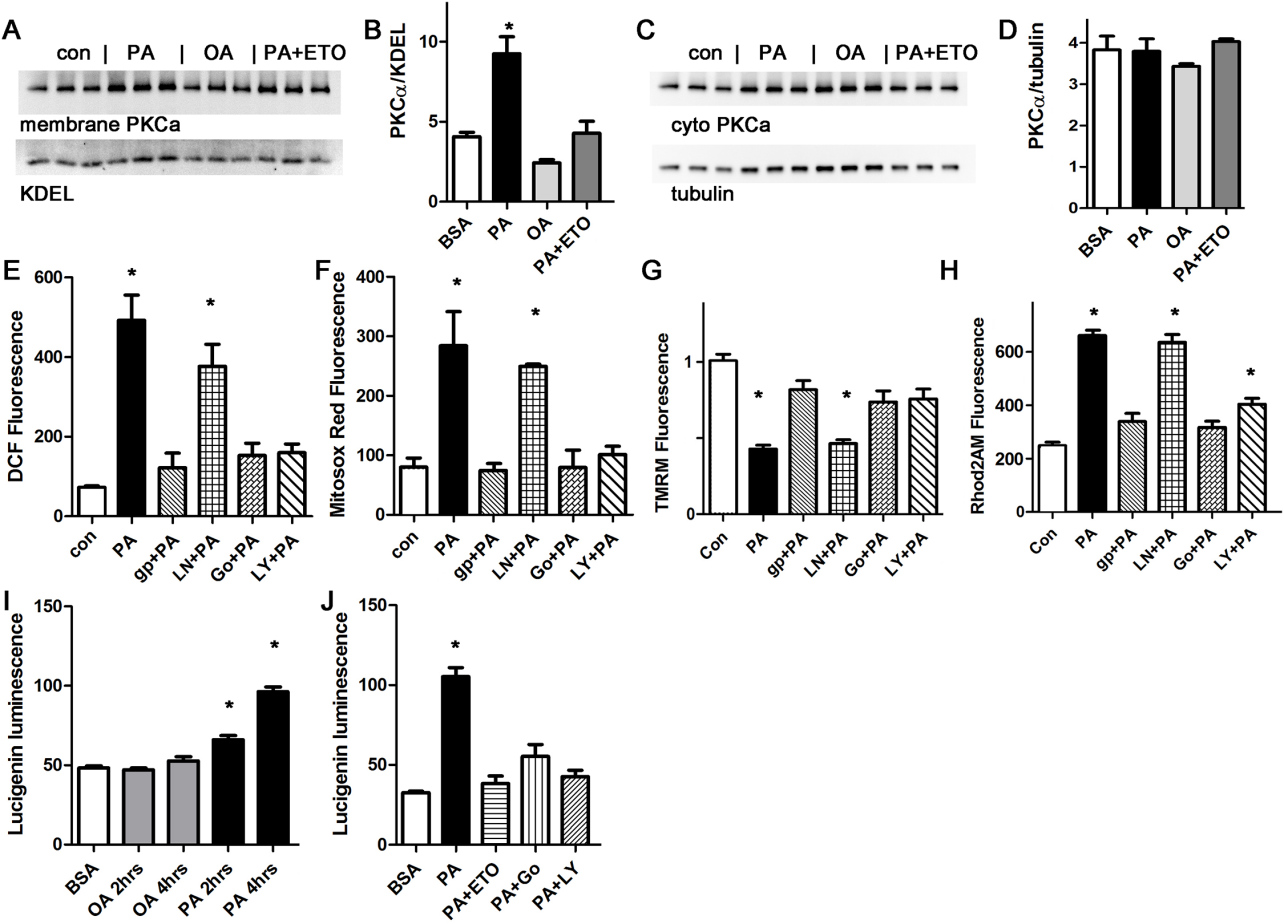
Palmitate activates PKC, and palmitate-induced ROS production is inhibited by blocking PKC or NOX2. A. Western blot from membrane preparation, arbitrary units, H9c2 cells. KDEL is a loading control for sarcoplasmic reticulum. B. Graph of membrane protein band quantification from panel A, expressed in arbitrary units. C. Western blot from cytosolic fraction, arbitrary units, H9c2 cells. D. Graph of membrane protein band quantification from panel C. No significant difference by ANOVA. E. Representative experiment done with cardiomyocytes in triplicate, height is DCF fluorescence minus background, in live cells, mean + SEM. F. Cardiomyocytes from the same experiment using mitosox red readout. G. Cardiomyocytes from the same experiment using TMRM signal. H. Cardiomyocytes from the same experiment using Rhod2 signal. I. Time course of NOX2 activation with oleate or palmitate, using H9c2 cells, expressed in arbitrary units. J. Etomoxir and PKC inhibitors prevent NOX2 activation, representative experiment using H9c2 cells. For all panels except D, means are significantly different by ANOVA, * = sig different from control by Dunnett post-hoc test. PA = palmitate 200 μM, OA = oleate 200 μM, ETO = etomoxir 200 gp = gp91ds peptide 50 μM, Ln = L-NAME 10 μM, Go = Go6983 5 μM, LY = LY333531 50 nM.

Lipids are known to activate conventional and novel PKC isoforms. To measure PKC activation, cardiomyocytes were treated with palmitate and oleate, and then harvested for membrane preparation western blots. PKCalpha was activated by palmitate, but not by oleate (Fig 4A and 4B). To determine if palmitate-induced ROS requires PKC activation, we inhibited PKC with two different pharmacologic inhibitors, Go6983 (a broad-spectrum PKC inhibitor) and LY333531 (aka ruboxistaurin, thought to be more specific for PKCalpha and beta [37]). Both PKC inhibitors resulted in a significant decrease in total cellular and mitochondrial ROS, demonstrating that PKC activation is necessary for palmitate-induced ROS generation (Fig 4C and 4D). Further, the PKC inhibitors also prevented the decrease in mitochondrial inner membrane potential and the mitochondrial calcium overload induced by palmitate (Fig 4E and 4F).

In other cells types, PKC activation increases ROS by activating NOX2 [38]. We found that the NOX2 inhibitor apocynin was effective at preventing the increase in total cellular and mitochondrial ROS in cardiomyocytes (S2 file). We also used a NOX2 inhibitor peptide, gp91ds-tat, to ensure specificity. The peptide inhibitor prevented the increase in ROS as detected by DCF and mitosox red (Fig 4C and 4D). The peptide inhibitor also prevented mitochondrial dysfunction, by preventing depolarization and calcium overload (Fig 4E and 4F). L-NAME, a specific inhibitor of nitric oxide synthase, which is another source of ROS in cardiomyocytes, had minimal effect. Thus, NOX2 activation is required for the palmitate-induced increase in ROS, and nitric oxide synthase is not involved. We also repeated these experiments using the H9c2 cell line. These results were consistent (S3 file). This shows that palmitate-induced mitochondrial dysfunction requires both PKC and NOX2 activation. In cardiomyocytes, it appears that NOX2 activation amplifies mitochondrial ROS production in a feed-forward cycle, an example of the phenomenon known as ROS induced ROS release (RIRR) [39].

To improve our understanding of the mechanisms involved in NOX2 activation, we used the lucigenin assay to measure NOX2 activity [32]. Lucigenin consistently showed a significant increase in NOX2 activity after two hours of palmitate treatment (Fig 4I and 4J). The increase in NOX2 activity was prevented by inhibition of mitochondrial uptake of fatty acids using etomoxir, and also by the PKC inhibitors, Go6983 and LY333531. This is consistent with a mechanism where mitochondrial uptake of palmitate causes a small initial increase in mitochondrial ROS, which activates PKC, which in turn activates NOX2.

### PKC-induced mitochondrial ROS is generated by NOX2 in cardiomyocytes

**Fig 5:**
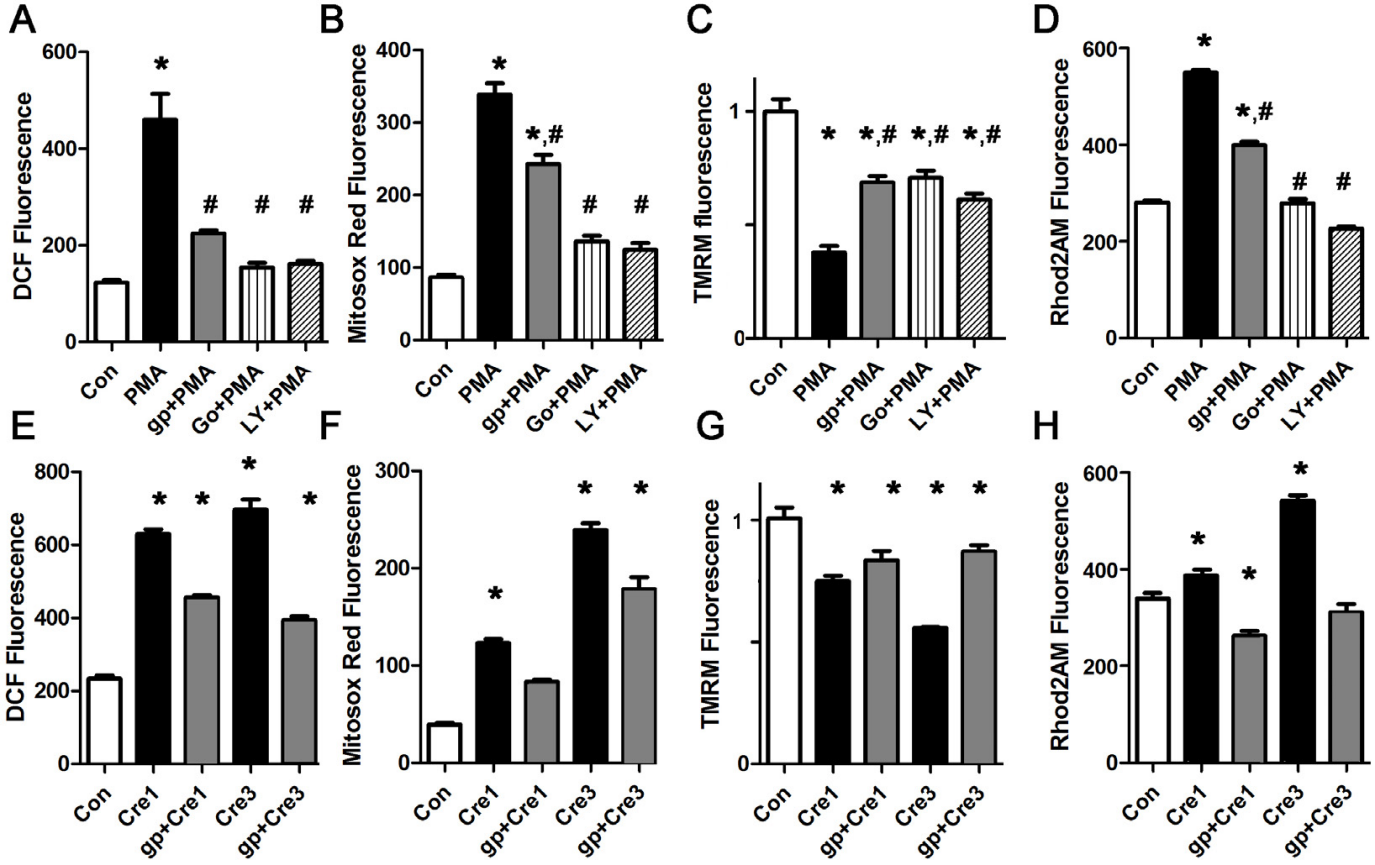
NOX2 inhibition reduces PMA-induced and cresol-induced ROS. A. Representative PMA experiment done with cardiomyocytes in triplicate, height is DCF fluorescence minus background, in live cells, expressed in arbitrary units, mean + SEM. B. PMA experiment using mitosox red signal. C. PMA experiment using TMRM signal. The NOX2 inhibitor and PKC inhibitors reduce depolarization. D. PMA experiment using Rhod2 signal. The NOX2 inhibitor and PKC inhibitors reduce calcium overload. E,F. Cresol increases total ROS and mitochondrial ROS. G. Cresol depolarized the mitochondrial inner membrane. H. Cresol increases mitochondrial calcium. For all panels, means are significantly different by ANOVA, * = sig different from control by post-hoc test, # = sig different from PMA by post-hoc test. PMA 100 nM, gp = gp91ds peptide 50 μM, Go = Go6983 5 μM, LY = LY333531 50 nM, Cre = cresol 1 or 3 μM.

To determine how PKC activation causes ROS generation in cardiomyocytes, we treated cardiomyocytes with the PKC activator PMA, which is known to increase ROS in several cell types. After one hour, PMA caused a significant increase in total ROS and mitochondrial ROS (Fig 5A and 5B). Pharmacologic inhibition of NOX2 was almost as effective in preventing the increase in DCF signal as PKC inhibition, demonstrating that PKC activation increases total cellular ROS predominantly via NOX2 in cardiomyocytes. PMA also caused depolarization of the mitochondrial inner membrane potential and mitochondrial calcium overload, and these abnormalities are partially prevented by the NOX2 inhibitor (Fig 5C and 5D). This shows that NOX2 activation is a major mechanism of PKC-activation induced ROS and mitochondrial dysfunction in cardiomyocytes.

Additional experiments showed that mitotempo improved the abnormalities caused by PMA (S4 file). This is consistent with a mechanism where mitochondrial ROS amplifies NOX2 ROS production in a feed-forward cycle.

### Increasing sarcoplasmic reticulum calcium leak is sufficient to generate mitochondrial ROS that is amplified by NOX2, leading to greater mitochondrial dysfunction

To determine if increasing RyR calcium release is sufficient to cause mitochondrial depolarization and ROS, we treated cardiomyocytes with cresol (aka 4-chloro-3-methylphenol) which is a well-described activator of RyR channels [40]. At moderate doses, cresol caused a significant increase in both total ROS and mitochondrial ROS (Fig 5E and 5F). Mitochondrial inner membrane potential was decreased by increased RyR leak (Fig 5G). These abnormalities were partially improved by the NOX2 inhibitor peptide. We confirmed that cresol caused mitochondrial calcium overload, which was improved by the NOX2 inhibitor peptide (Fig 5H). Thus, even in this relatively pure case of sarcoplasmic reticulum calcium leak, NOX2 activation amplifies mitochondrial ROS, causing worse mitochondrial depolarization and calcium overload.

### NOX2 KO cardiomyocytes do not generate palmitate-induced ROS, or PKC-induced ROS

**Fig 6:**
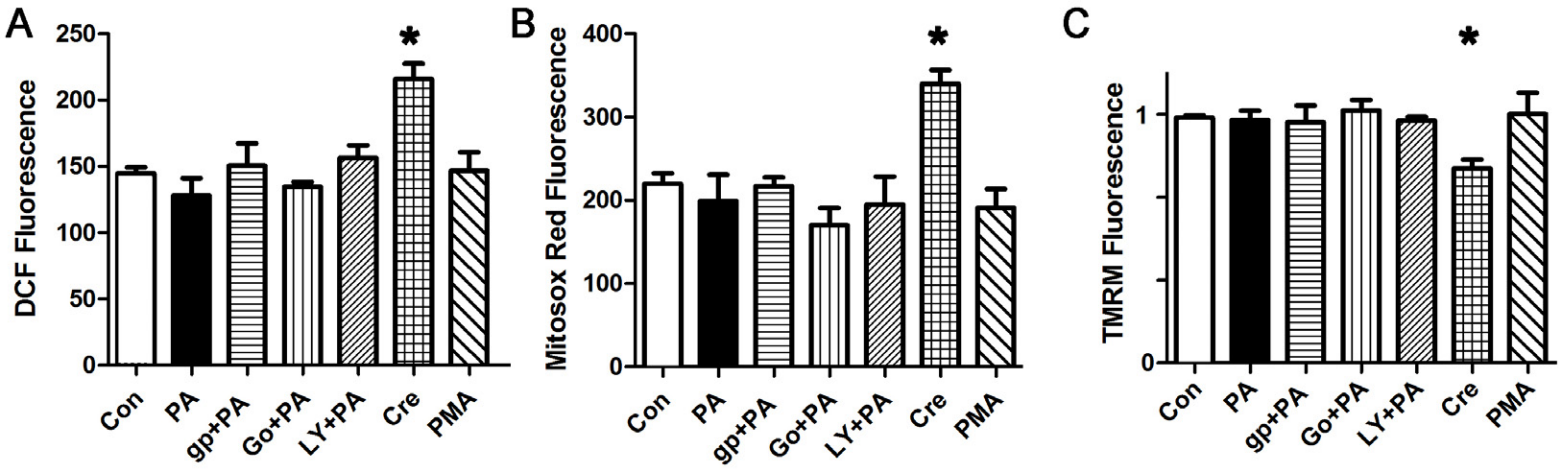
NOX2 KO cardiomyocytes do not have an increase in ROS in response to palmitate or PKC activation. A. Representative experiment done with cardiomyocytes in triplicate, height is DCF fluorescence minus background, in live cells, expressed in arbitrary units, mean + SEM. B. Cardiomyocytes from the same experiment using mitosox red readout. C. Cardiomyocytes from the same experiment using TMRM readout. For all panels, means are significantly different by ANOVA, * = sig different from control by Dunnett post-hoc test. PA = palmitate 200 μM, gp = gp91ds peptide 50 μM, Go = Go6983 5 μM, LY = LY333531 50 nM, Cre = cresol 3 μM, PMA 100 nM.

To confirm the specificity of our findings with apocynin and the NOX2 inhibitor peptide, we pursued a genetic approach. NOX2 is composed of p47phox (which is also a component of NOX1), the catalytic protein gp91, and several regulatory subunits. We used isolated cardiomyocytes from adult male NOX2 KO mice, and determined that palmitate does not increase total or mitochondrial ROS in NOX2 KO cardiomyocytes (Fig 6A and 6B). Further, NOX2 KO cardiomyocytes did not have mitochondrial depolarization or mitochondrial calcium overload after palmitate treatment. This proves that NOX2 activity is required for palmitate-induced ROS generation and mitochondrial dysfunction. Treatment of the NOX2 KO cardiomyocytes with PMA, to activate endogenous PKC, did not result in ROS, mitochondrial depolarization or mitochondrial calcium overload. This is additional evidence that PKC generates ROS predominantly by activating NOX2 in cardiomyocytes. As a positive control, cresol still caused an increase in ROS and depolarized the mitochondrial inner membrane in NOX2 KO cardiomyocytes, though the magnitude of the effect was less than the abnormalities induced in WT cardiomyocytes. This is consistent with increased RyR leak causing calcium overload of mitochondria, leading to mild mitochondrial ROS generation that is then amplified by NOX2 activation in WT cardiomyocytes.

### Blockade of the mitochondrial electron transport chain causes ROS and mitochondrial inner membrane depolarization that is similar to palmitate

**Fig 7:**
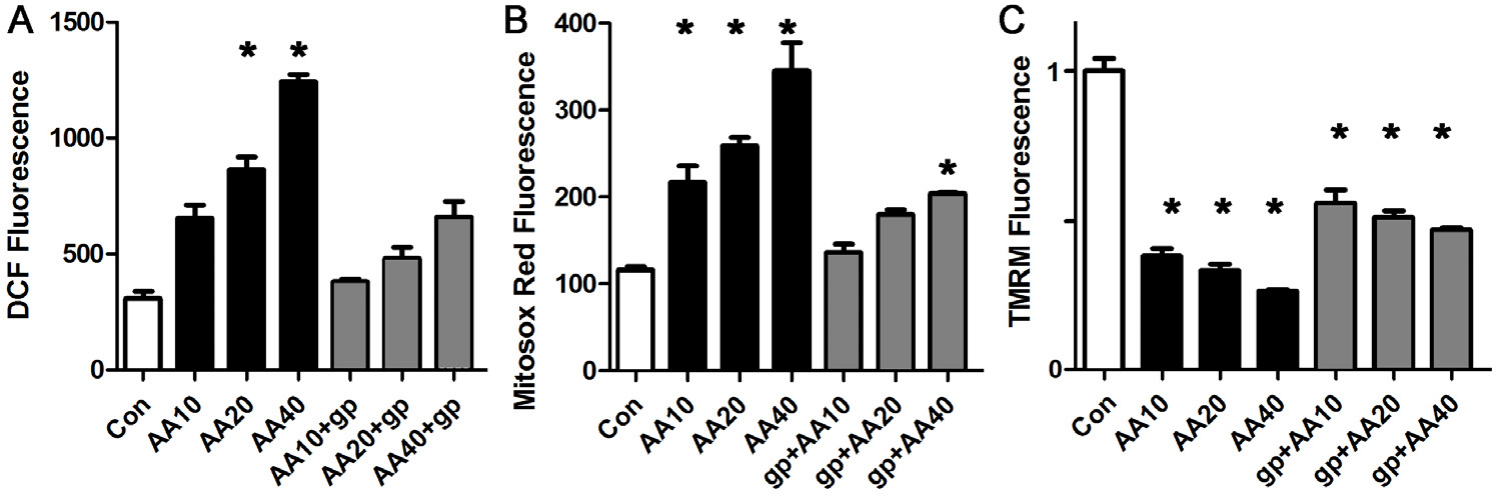
Blocking electron transport chain with antimycin-A causes ROS and mitochondrial depolarization. A. Representative experiment done with cardiomyocytes in triplicate, height is DCF fluorescence minus background, in live cells, mean + SEM expressed in arbitrary units. B. Cardiomyocytes from the same experiment using mitosox red readout. C. Cardiomyocytes from the same experiment using TMRM signal. For all panels, means are significantly different by ANOVA, * = sig different from control by post-hoc test. gp = gp91ds peptide 50 μM, AA = antimycin-A at 10, 20 or 40 μM.

Fatty acids are thought to act as mitochondrial uncouplers, but have also been postulated to block the mitochondrial electron transport chain [26, 41–43]. To determine if the observed palmitate-induced abnormalities are consistent with the block mechanism, we performed a series of experiments with antimycin-A, which selectively blocks complex III of the electron transport chain. Escalating doses of antimycin-A caused a step-wise decrease in the mitochondrial inner membrane potential and resulted in a significant increase in total ROS and mitochondrial ROS in cardiomyocytes (Fig 7). The NOX2 inhibitor peptide prevented the increase in total ROS, and blunted the increase in mitochondrial ROS, without having much effect on the reduction in mitochondrial inner membrane potential. This demonstrates that the abnormalities induced by palmitate are similar to the abnormalities induced by partially blocking the electron transport chain. The fact that the NOX2 inhibitor peptide had only a minor effect on the reduction in mitochondrial inner membrane potential is consistent with the direct effect of antimycin-A on the electron transport chain. These findings also demonstrate that a reduction in mitochondrial inner membrane potential is not necessarily associated with a significant increase in ROS, if NOX2 is inhibited.

## Discussion

**Fig 8:**
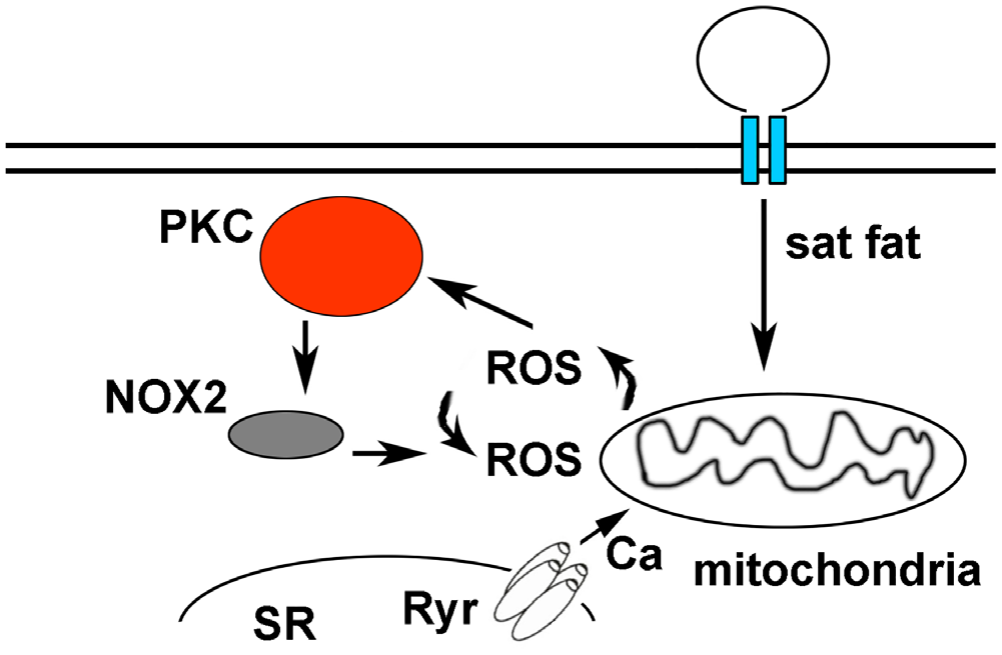
Diagram of proposed pathway. Saturated fats are internalized by cardiomyocytes and transported to the mitochondria to be used as a fuel source for beta-oxidation, causing a low level of mitochondrial ROS. This activates PKC, which in turn activates NOX2. ROS from NOX2 and mitochondrial ROS amplify each other in a feed-forward cycle that promotes greater ROS production and sarcoplasmic reticulum (SR) calcium leak.

Our findings advance the understanding of cardiac biology by demonstrating that a physiologic level of saturated fat inhibit mitochondrial oxidative phosphorylation and cause ROS in cardiomyocytes. Palmitate causes mitochondrial ROS that is amplified by PKC-NOX2 activation, leading to abnormal calcium homeostasis (Fig 8). By using pharmacologic inhibition of NOX2 and cardiomyocytes from NOX2 KO mice, we show that NOX2 has a central role in the generation of ROS caused by saturated fat. Furthermore, our studies identify a molecular mechanism by which saturated fats can promote heart failure and arrhythmia.

### Palmitate, mitochondria, and the heart

Although cardiac mitochondria have been the focus of scientific research for decades, much less is known about mitochondrial fatty-acid metabolism and ROS generation in intact cardiomyocytes. By using intact cells, we have been able to study cellular pathways that would be disturbed in permeabilized cells or purified mitochondrial preparations. Palmitate is a normal part of the human diet, yet there is strong evidence that it can cause multiple abnormalities. High doses of palmitate can induce apoptosis in vitro [44, 45]. Palmitate-induced ROS has also been implicated in insulin resistance [23]. Our data, showing a decrease in maximum oxidative phosphorylation, in combination with decreased inner membrane potential, is consistent with palmitate acting as a partial inhibitor of the electron transport chain. Direct measures of mitochondrial complex activity support this mechanism. Using the isolated cardiomyocytes model system, we show that partial inhibition of the electron transport chain causes abnormalities that are similar to palmitate, supporting this mode of action. These mitochondrial abnormalities may contribute to the increased risk of heart failure arrhythmia in obese and diabetic patients, since these diseases cause cardiac lipid overload.

### PKC, NOX, and ROS

It is possible that palmitate can cause low-level activation of PKC directly, which leads to NOX2 activation. However, the experiments with etomoxir, shown in Fig 4, indicate that full activation of PKC requires palmitate to enter the mitochondria. We postulate that palmitate initially causes a small amount of mitochondrial ROS. This activates NOX2 via PKC, generating more ROS, which causes more mitochondrial dysfunction in a feed-forward cycle. In vascular tissue, there is cross talk between mitochondrial ROS and NOX, such that the one can amplify the other [38]. ROS-induced ROS release (RIRR) has been shown to occur in cardiomyocytes using stimulation such as photo-induced oxidation [46] and in Langendorff preparations perfused with H_2_O_2_ [39]. The molecular pathways responsible for RIRR in the heart have not been identified. We demonstrate that is critical for amplifying mitochondrial ROS in cardiomyocytes, and thus NOX2 is a critical component of RIRR in this model system. The role of NOX enzymes in cardiac pathology is complex [47]. Low-level NOX activity may be beneficial and generate ROS as signaling intermediates. However, NOX activity is increased in human heart failure [48] and NOX2 is activated in the atria of patients with atrial fibrillation and large animal models of atrial fibrillation [49, 50]. Chronic activation of NOX2 by saturated fat could promote heart failure and arrhythmia.

We also show that NOX2 is required for ROS generation caused by PKC activation in cardiomyocytes. Further, we show that activating endogenous PKC in cardiomyocytes is sufficient to trigger NOX2-mediated ROS generation that in turn causes an increase in mitochondrial ROS. Our experiments identify NOX2 as the major mediator of PKC induced ROS in cardiomyocytes. Activation of conventional PKC isoforms has been implicated as a major factor in heart failure pathophysiology [51]. Additional work will be necessary to determine if of PKC’s harmful effects are mediated by NOX2-generated ROS in other disease states.

### Abnormal calcium homeostasis

The observation that saturated fat increases calcium sparks is novel. The interaction between NOX2 and RyR2 has been described previously, and accounts for increased sarcoplasmic reticulum release with myocytes stretch [52]. This interaction was not previously known to have a role in mitochondrial metabolic abnormalities. Our findings indicate that palmitate causes mitochondrial calcium overload due to increased sarcoplasmic reticulum leak from RyR channels. Since cardiac mitochondria are physically connected to the sarcoplasmic reticulum, and mitochondria take up calcium released from the sarcoplasmic reticulum [53], it is plausible that abnormal calcium release induced by palmitate could initiate mitochondrial dysfunction in cardiomyocytes. Inhibition of RyR calcium release prevents ROS production by preventing mitochondrial calcium overload and dysfunction. Our experiments with cresol demonstrate that increased sarcoplasmic reticulum leak is sufficient to trigger mitochondrial ROS. These results give us mechanistic insight into the cardiac biology that connects lipid overload to abnormal calcium homeostasis and mitochondrial function.

We conclude that, in adult cardiomyocytes, palmitate induces ROS by a mechanism that requires mitochondrial uptake and beta-oxidation of palmitate, and the mitochondrial ROS is amplified by PKC-NOX2 activation. Activating this pathway results in increased sarcoplasmic reticulum calcium leak, mitochondrial calcium overload, and mitochondrial dysfunction. Pharmacologic NOX2 inhibition prevents the abnormalities caused by saturated fat. The mechanisms revealed by this work may have therapeutic implication for heart disease caused by diabetes and obesity. Although clinical trials of antioxidants for cardiovascular disease have been disappointing, for the most part, there is some clinical evidence supporting beneficial effects on heart rhythm [54–56]. It may be that antioxidants are only beneficial in certain forms of cardiovascular disease, where increased ROS has a central role in the pathophysiology. Pharmacotherapy that specifically targets the source of ROS could be more effective. NOX2 inhibition may be a potential therapy for heart rhythm abnormalities in obese and diabetic patients. In addition, since increased RyR calcium leak is an important mechanism in heart failure [57], it is possible that NOX2 inhibition could be used as a treatment for heart failure.

## Supporting Information

S1 File H9c2 treated with palmitate have increased total and mitochondrial ROS. (DOCX)

S2 File Apocynin prevents PA induced ROS in cardiomyocytes. (DOCX)

S3 File PA activates PKC and PA-induced ROS production is inhibited by blocking NOX2 or PKC in H9c2 cells. (DOCX)

S4 File Mitotempo improves PMA-induced ROS in cardiomyocytes. (DOCX)

## References

[1] Szczepaniak LS, Victor RG, Orci L, Unger RH. Forgotten but not gone: the rediscovery of fatty heart, the most common unrecognized disease in America. Circ Res. 2007;101:759–67. 17932333 10.1161/CIRCRESAHA.107.160457

[2] Morrow JP, Katchman A, Son NH, Trent CM, Khan R, Shiomi T, et al Mice With Cardiac Overexpression of Peroxisome Proliferator-Activated Receptor gamma Have Impaired Repolarization and Spontaneous Fatal Ventricular Arrhythmias. Circulation. 2011;124:2812–21. 10.1161/CIRCULATIONAHA.111.056309 22124376 PMC3258098

[3] Son NH, Park TS, Yamashita H, Yokoyama M, Huggins LA, Okajima K, et al Cardiomyocyte expression of PPARgamma leads to cardiac dysfunction in mice. J Clin Invest. 2007;117:2791–801. 17823655 10.1172/JCI30335 PMC1964508

[4] Sharma S, Adrogue JV, Golfman L, Uray I, Lemm J, Youker K, et al Intramyocardial lipid accumulation in the failing human heart resembles the lipotoxic rat heart. FASEB J. 2004;18:1692–700. 15522914 10.1096/fj.04-2263com

[5] Selvin E, Lazo M, Chen Y, Shen L, Rubin J, McEvoy JW, et al Diabetes, Pre-Diabetes and Incidence of Subclinical Myocardial Damage. Circulation. 2014. 10.1161/CIRCULATIONAHA.114.010815 PMC4198442 25149362

[6] Kenchaiah S, Evans JC, Levy D, Wilson PW, Benjamin EJ, Larson MG, et al Obesity and the risk of heart failure. The New England journal of medicine. 2002;347:305–13. 12151467 10.1056/NEJMoa020245

[7] Kannel WB, Hjortland M, Castelli WP. Role of diabetes in congestive heart failure: the Framingham study. Am J Cardiol. 1974;34:29–34. 4835750 10.1016/0002-9149(74)90089-7

[8] Wang TJ, Parise H, Levy D, D'Agostino RB Sr., Wolf PA, Vasan RS, et al Obesity and the risk of new-onset atrial fibrillation. Jama. 2004;292:2471–7. 15562125 10.1001/jama.292.20.2471

[9] Alonso A, Krijthe BP, Aspelund T, Stepas KA, Pencina MJ, Moser CB, et al Simple risk model predicts incidence of atrial fibrillation in a racially and geographically diverse population: the CHARGE-AF consortium. Journal of the American Heart Association. 2013;2:e000102 10.1161/JAHA.112.000102 23537808 PMC3647274

[10] Albert CM, Chae CU, Grodstein F, Rose LM, Rexrode KM, Ruskin JN, et al Prospective study of sudden cardiac death among women in the United States. Circulation. 2003;107:2096–101. 12695299 10.1161/01.CIR.0000065223.21530.11

[11] Jouven X, Desnos M, Guerot C, Ducimetiere P. Predicting sudden death in the population: the Paris Prospective Study I. Circulation. 1999;99:1978–83. 10209001 10.1161/01.cir.99.15.1978

[12] Filippi A, Sessa E Jr., Mazzaglia G, Pecchioli S Jr., Capocchi R Jr., Caprari F, et al Out of hospital sudden cardiac death in Italy: a population-based case-control study. J Cardiovasc Med (Hagerstown). 2008;9:595–600. 18475128 10.2459/JCM.0b013e3282f2c9d0

[13] Hookana E, Junttila MJ, Puurunen VP, Tikkanen JT, Kaikkonen KS, Kortelainen ML, et al Causes of nonischemic sudden cardiac death in the current era. Heart Rhythm. 2011;8:1570–5. 10.1016/j.hrthm.2011.06.031 21740887

[14] Jouven X, Charles MA, Desnos M, Ducimetiere P. Circulating nonesterified fatty acid level as a predictive risk factor for sudden death in the population. Circulation. 2001;104:756–61. 11502698 10.1161/hc3201.094151

[15] Oliver MF, Kurien VA, Greenwood TW. Relation between serum-free-fatty acids and arrhythmias and death after acute myocardial infarction. Lancet. 1968;1:710–4. 4170959 10.1016/s0140-6736(68)92163-6

[16] Chiuve SE, Rimm EB, Sandhu RK, Bernstein AM, Rexrode KM, Manson JE, et al Dietary fat quality and risk of sudden cardiac death in women. The American journal of clinical nutrition. 2012;96:498–507. 10.3945/ajcn.112.040287 22854398 PMC3417213

[17] Lemaitre RN, King IB, Sotoodehnia N, Knopp RH, Mozaffarian D, McKnight B, et al Endogenous red blood cell membrane fatty acids and sudden cardiac arrest. Metabolism. 2010;59:1029–34. 10.1016/j.metabol.2009.10.026 20045147 PMC2882498

[18] Luo M, Guan X, Luczak ED, Lang D, Kutschke W, Gao Z, et al Diabetes increases mortality after myocardial infarction by oxidizing CaMKII. J Clin Invest. 2013;123:1262–74. 10.1172/JCI65268 23426181 PMC3673230

[19] Yang KC, Dudley SC Jr. Oxidative stress and atrial fibrillation: finding a missing piece to the puzzle. Circulation. 2013;128:1724–6. 10.1161/CIRCULATIONAHA.113.005837 24030497 PMC3909513

[20] Steinberg SF. Oxidative stress and sarcomeric proteins. Circ Res. 2013;112:393–405. 10.1161/CIRCRESAHA.111.300496 23329794 PMC3595003

[21] Santos CX, Anilkumar N, Zhang M, Brewer AC, Shah AM. Redox signaling in cardiac myocytes. Free Radic Biol Med. 2011;50:777–93. 10.1016/j.freeradbiomed.2011.01.003 21236334 PMC3049876

[22] Madamanchi NR, Runge MS. Redox signaling in cardiovascular health and disease. Free Radic Biol Med. 2013;61C:473–501. 10.1016/j.freeradbiomed.2013.04.001 PMC3883979 23583330

[23] Anderson EJ, Lustig ME, Boyle KE, Woodlief TL, Kane DA, Lin CT, et al Mitochondrial H2O2 emission and cellular redox state link excess fat intake to insulin resistance in both rodents and humans. J Clin Invest. 2009;119:573–81. 10.1172/JCI37048 19188683 PMC2648700

[24] Cole MA, Murray AJ, Cochlin LE, Heather LC, McAleese S, Knight NS, et al A high fat diet increases mitochondrial fatty acid oxidation and uncoupling to decrease efficiency in rat heart. Basic Res Cardiol. 2011;106:447–57. 10.1007/s00395-011-0156-1 21318295 PMC3071466

[25] St-Pierre J, Buckingham JA, Roebuck SJ, Brand MD. Topology of superoxide production from different sites in the mitochondrial electron transport chain. J Biol Chem. 2002;277:44784–90. 12237311 10.1074/jbc.M207217200

[26] Schonfeld P, Wojtczak L. Fatty acids decrease mitochondrial generation of reactive oxygen species at the reverse electron transport but increase it at the forward transport. Biochim Biophys Acta. 2007;1767:1032–40. 17588527 10.1016/j.bbabio.2007.04.005

[27] Koves TR, Ussher JR, Noland RC, Slentz D, Mosedale M, Ilkayeva O, et al Mitochondrial overload and incomplete fatty acid oxidation contribute to skeletal muscle insulin resistance. Cell Metab. 2008;7:45–56. 10.1016/j.cmet.2007.10.013 18177724

[28] Micha R, Mozaffarian D. Saturated fat and cardiometabolic risk factors, coronary heart disease, stroke, and diabetes: a fresh look at the evidence. Lipids. 2010;45:893–905. 10.1007/s11745-010-3393-4 20354806 PMC2950931

[29] Watt MJ, Hoy AJ, Muoio DM, Coleman RA. Distinct roles of specific fatty acids in cellular processes: implications for interpreting and reporting experiments. Am J Physiol Endocrinol Metab. 2012;302:E1–3. 10.1152/ajpendo.00418.2011 22180647 PMC3774556

[30] Eruslanov E, Kusmartsev S. Identification of ROS using oxidized DCFDA and flow-cytometry. Methods Mol Biol. 2010;594:57–72. 10.1007/978-1-60761-411-1_4 20072909

[31] Bowser DN, Minamikawa T, Nagley P, Williams DA. Role of mitochondria in calcium regulation of spontaneously contracting cardiac muscle cells. Biophysical journal. 1998;75:2004–14. 9746542 10.1016/S0006-3495(98)77642-8 PMC1299872

[32] Bendall JK, Cave AC, Heymes C, Gall N, Shah AM. Pivotal role of a gp91(phox)-containing NADPH oxidase in angiotensin II-induced cardiac hypertrophy in mice. Circulation. 2002;105:293–6. 11804982 10.1161/hc0302.103712

[33] DiMauro S, Servidei S, Zeviani M, DiRocco M, DeVivo DC, DiDonato S, et al Cytochrome c oxidase deficiency in Leigh syndrome. Annals of neurology. 1987;22:498–506. 2829705 10.1002/ana.410220409

[34] Birch-Machin MA, Briggs HL, Saborido AA, Bindoff LA, Turnbull DM. An evaluation of the measurement of the activities of complexes I-IV in the respiratory chain of human skeletal muscle mitochondria. Biochemical medicine and metabolic biology. 1994;51:35–42. 8192914 10.1006/bmmb.1994.1004

[35] Brynes AE, Mark Edwards C, Ghatei MA, Dornhorst A, Morgan LM, Bloom SR, et al A randomised four-intervention crossover study investigating the effect of carbohydrates on daytime profiles of insulin, glucose, non-esterified fatty acids and triacylglycerols in middle-aged men. The British journal of nutrition. 2003;89:207–18. 12575905 10.1079/BJN2002769

[36] Brand MD, Nicholls DG. Assessing mitochondrial dysfunction in cells. Biochem J. 2011;435:297–312. 10.1042/BJ20110162 21726199 PMC3076726

[37] Liu Q, Chen X, Macdonnell SM, Kranias EG, Lorenz JN, Leitges M, et al Protein kinase C{alpha}, but not PKC{beta} or PKC{gamma}, regulates contractility and heart failure susceptibility: implications for ruboxistaurin as a novel therapeutic approach. Circ Res. 2009;105:194–200. 10.1161/CIRCRESAHA.109.195313 19556521 PMC2749656

[38] Dikalov S. Cross talk between mitochondria and NADPH oxidases. Free Radic Biol Med. 2011;51:1289–301. 10.1016/j.freeradbiomed.2011.06.033 21777669 PMC3163726

[39] Biary N, Xie C, Kauffman J, Akar FG. Biophysical properties and functional consequences of reactive oxygen species (ROS)-induced ROS release in intact myocardium. J Physiol. 2011;589:5167–79. 10.1113/jphysiol.2011.214239 21825030 PMC3225672

[40] Fessenden JD, Feng W, Pessah IN, Allen PD. Amino acid residues Gln4020 and Lys4021 of the ryanodine receptor type 1 are required for activation by 4-chloro-m-cresol. J Biol Chem. 2006;281:21022–31. 16737973 10.1074/jbc.M600670200

[41] Carlsson C, Borg LA, Welsh N. Sodium palmitate induces partial mitochondrial uncoupling and reactive oxygen species in rat pancreatic islets in vitro. Endocrinology. 1999;140:3422–8. 10433196 10.1210/endo.140.8.6908

[42] Boudina S, Sena S, O'Neill BT, Tathireddy P, Young ME, Abel ED. Reduced mitochondrial oxidative capacity and increased mitochondrial uncoupling impair myocardial energetics in obesity. Circulation. 2005;112:2686–95. 16246967 10.1161/CIRCULATIONAHA.105.554360

[43] Wojtczak L, Schonfeld P. Effect of fatty acids on energy coupling processes in mitochondria. Biochim Biophys Acta. 1993;1183:41–57. 8399375 10.1016/0005-2728(93)90004-y

[44] Dyntar D, Eppenberger-Eberhardt M, Maedler K, Pruschy M, Eppenberger HM, Spinas GA, et al Glucose and palmitic acid induce degeneration of myofibrils and modulate apoptosis in rat adult cardiomyocytes. Diabetes. 2001;50:2105–13. 11522678 10.2337/diabetes.50.9.2105

[45] Listenberger LL, Han X, Lewis SE, Cases S, Farese RV Jr., Ory DS, et al Triglyceride accumulation protects against fatty acid-induced lipotoxicity. Proc Natl Acad Sci U S A. 2003;100:3077–82. 12629214 10.1073/pnas.0630588100 PMC152249

[46] Zorov DB, Filburn CR, Klotz LO, Zweier JL, Sollott SJ. Reactive oxygen species (ROS)-induced ROS release: a new phenomenon accompanying induction of the mitochondrial permeability transition in cardiac myocytes. The Journal of experimental medicine. 2000;192:1001–14. 11015441 10.1084/jem.192.7.1001 PMC2193314

[47] Sirker A, Zhang M, Shah AM. NADPH oxidases in cardiovascular disease: insights from in vivo models and clinical studies. Basic Res Cardiol. 2011;106:735–47. 10.1007/s00395-011-0190-z 21598086 PMC3149671

[48] Heymes C, Bendall JK, Ratajczak P, Cave AC, Samuel JL, Hasenfuss G, et al Increased myocardial NADPH oxidase activity in human heart failure. J Am Coll Cardiol. 2003;41:2164–71. 12821241 10.1016/s0735-1097(03)00471-6

[49] Kim YM, Guzik TJ, Zhang YH, Zhang MH, Kattach H, Ratnatunga C, et al A myocardial Nox2 containing NAD(P)H oxidase contributes to oxidative stress in human atrial fibrillation. Circ Res. 2005;97:629–36. 16123335 10.1161/01.RES.0000183735.09871.61

[50] Dudley SC Jr., Hoch NE, McCann LA, Honeycutt C, Diamandopoulos L, Fukai T, et al Atrial fibrillation increases production of superoxide by the left atrium and left atrial appendage: role of the NADPH and xanthine oxidases. Circulation. 2005;112:1266–73. 16129811 10.1161/CIRCULATIONAHA.105.538108

[51] Braz JC, Gregory K, Pathak A, Zhao W, Sahin B, Klevitsky R, et al PKC-alpha regulates cardiac contractility and propensity toward heart failure. Nat Med. 2004;10:248–54. 14966518 10.1038/nm1000

[52] Prosser BL, Ward CW, Lederer WJ. X-ROS signaling: rapid mechano-chemo transduction in heart. Science. 2011;333:1440–5. 10.1126/science.1202768 21903813

[53] Hayashi T, Martone ME, Yu Z, Thor A, Doi M, Holst MJ, et al Three-dimensional electron microscopy reveals new details of membrane systems for Ca2+ signaling in the heart. J Cell Sci. 2009;122:1005–13. 10.1242/jcs.028175 19295127 PMC2720931

[54] Harling L, Rasoli S, Vecht JA, Ashrafian H, Kourliouros A, Athanasiou T. Do antioxidant vitamins have an anti-arrhythmic effect following cardiac surgery? A meta-analysis of randomised controlled trials. Heart. 2011;97:1636–42. 10.1136/heartjnl-2011-300245 21865202

[55] Rodrigo R, Korantzopoulos P, Cereceda M, Asenjo R, Zamorano J, Villalabeitia E, et al A Randomized Controlled Trial to Prevent Postoperative Atrial Fibrillation by Antioxidant Reinforcement. J Am Coll Cardiol. 2013. 10.1016/j.jacc.2013.07.014 23916928

[56] Rodrigo R, Korantzopoulos P, Cereceda M, Asenjo R, Zamorano J, Villalabeitia E, et al A randomized controlled trial to prevent post-operative atrial fibrillation by antioxidant reinforcement. J Am Coll Cardiol. 2013;62:1457–65. 10.1016/j.jacc.2013.07.014 23916928

[57] Marx SO, Marks AR. Dysfunctional ryanodine receptors in the heart: new insights into complex cardiovascular diseases. J Mol Cell Cardiol. 2013;58:225–31. 10.1016/j.yjmcc.2013.03.005 23507255 PMC4042628

