# Correction: Inhibition of NAPDH Oxidase 2 (NOX2) Prevents Oxidative Stress and Mitochondrial Abnormalities Caused by Saturated Fat in Cardiomyocytes

**DOI:** 10.1371/journal.pone.0174525

**Published:** 2017-03-20

**Authors:** Leroy C. Joseph, Emanuele Barca, Prakash Subramanyam, Michael Komrowski, Utpal Pajvani, Henry M. Colecraft, Michio Hirano, John P. Morrow

In the title of this article, the word “NAPDH” should be “NADPH.” The correct title is: Inhibition of NADPH Oxidase 2 (NOX2) Prevents Oxidative Stress and Mitochondrial Abnormalities Caused by Saturated Fat in Cardiomyocytes. The correct citation is: Joseph LC, Barca E, Subramanyam P, Komrowski M, Pajvani U, Colecraft HM, et al. (2016) Inhibition of NADPH Oxidase 2 (NOX2) Prevents Oxidative Stress and Mitochondrial Abnormalities Caused by Saturated Fat in Cardiomyocytes. PLoS ONE 11(1): e0145750. doi:10.1371/journal.pone.0145750

The acronym NADPH appears incorrectly throughout the article. The correct acronym should be NADPH.
