# Supplementary material for: Inhibition of NAPDH Oxidase 2 (NOX2) Prevents Oxidative Stress and Mitochondrial Abnormalities Caused by Saturated Fat in Cardiomyocytes
Source: PLoS One. 2016 Jan 12;11(1):e0145750. doi: 10.1371/journal.pone.0145750 (PMC4710525; doi:10.1371/journal.pone.0145750)
Supplement: S1 File — (DOCX) [file pone.0145750.s001.docx]

**S1 file: Online Supplement for: Inhibition of NAPDH oxidase 2 (NOX2) prevents oxidative stress and mitochondrial abnormalities caused by saturated fat in cardiomyocytes**

**Supplemental Figure 1. H9c2 treated with palmitate have increased total and mitochondrial ROS.**

A. Representative experiment done with H9c2 in triplicate, height is DCF fluorescence minus background, in live cells, mean + SEM. Oleate does not increase ROS significantly. PA= palmitate 200 μM, OA= oleate 200 μM,

B. Parallel experiment in H9c2 cells using mitosox red readout to indicate mitochondrial ROS. C. H9c2 cells, inhibition of mitochondrial lipid uptake or beta-oxidation prevents the increase in ROS. ETO=etomoxir 200 μM, TMZ = trimetazidine 10 μM, MT= mito-TEMPO, 20 μM. D. Parallel experiment in H9c2 cells using mitosox red readout.

For panels A-D, means are significantly different by ANOVA, *= sig different from control by post-hoc test

E. Cell death quantified with trypan blue staining, H9c2 cells in triplicate at 4-hour exposure to indicated concentration of PA in μM. Background reading from unstained cells was subtracted from measurements. The difference in means is not significant by ANOVA

**
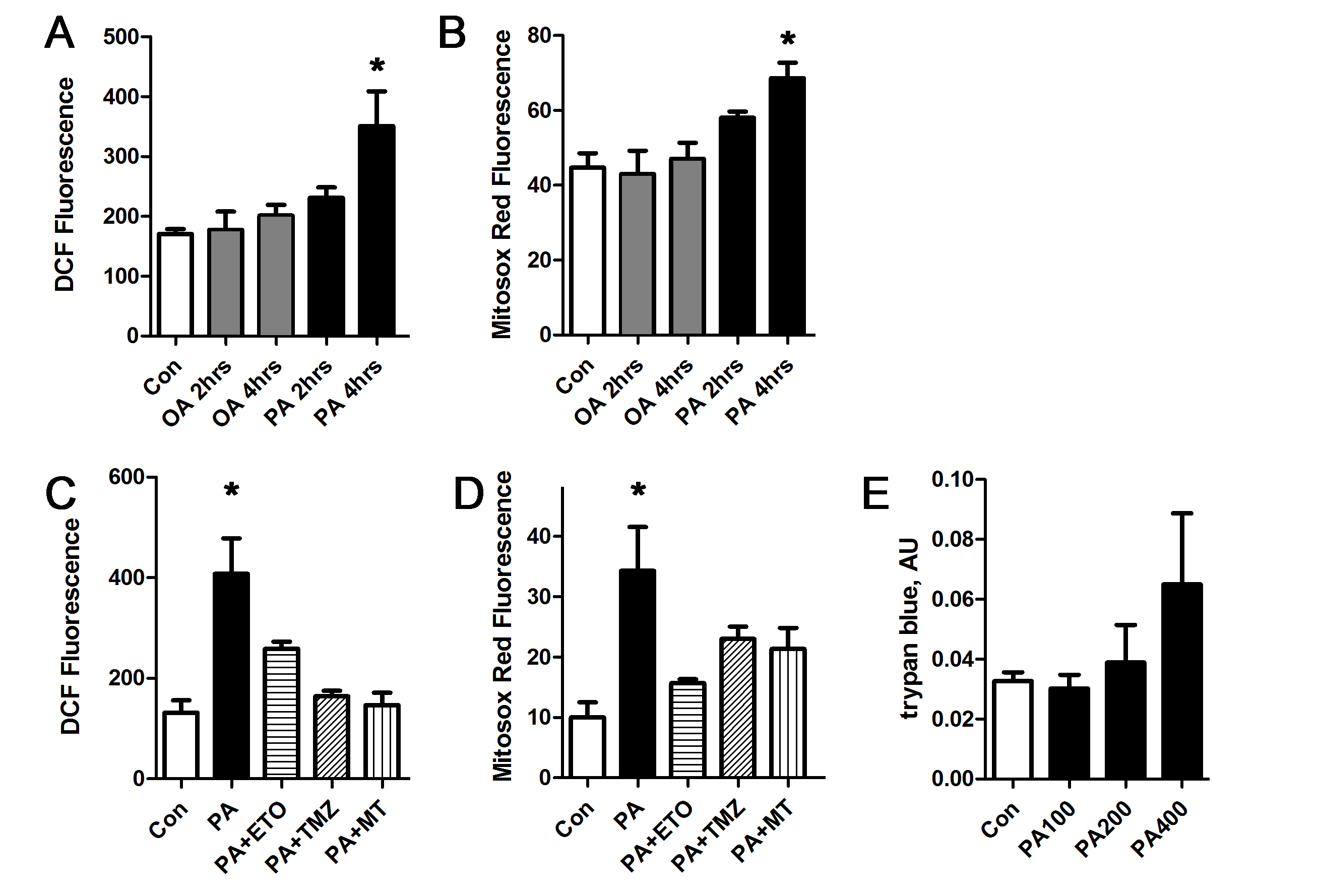
**
