# Supplementary material for: Inhibition of NAPDH Oxidase 2 (NOX2) Prevents Oxidative Stress and Mitochondrial Abnormalities Caused by Saturated Fat in Cardiomyocytes
Source: PLoS One. 2016 Jan 12;11(1):e0145750. doi: 10.1371/journal.pone.0145750 (PMC4710525; doi:10.1371/journal.pone.0145750)

**S2 file: Online Supplement for: Inhibition of NAPDH oxidase 2 (NOX2) prevents oxidative stress and mitochondrial abnormalities caused by saturated fat in cardiomyocytes**

# Supplemental Figure 2. Apocynin prevents PA induced ROS in cardiomyocytes

A. Representative experiment done with cardiomyocytes in triplicate, height is DCF fluorescence minus background, in live cells, mean + SEM. PA= palmitate 200 μM, Apo= apocynin 200 μM.

B. Cardiomyocytes from the same experiment using mitosox red readout. For both panels, means are significantly different by ANOVA, *= sig different from control by post-hoc test.


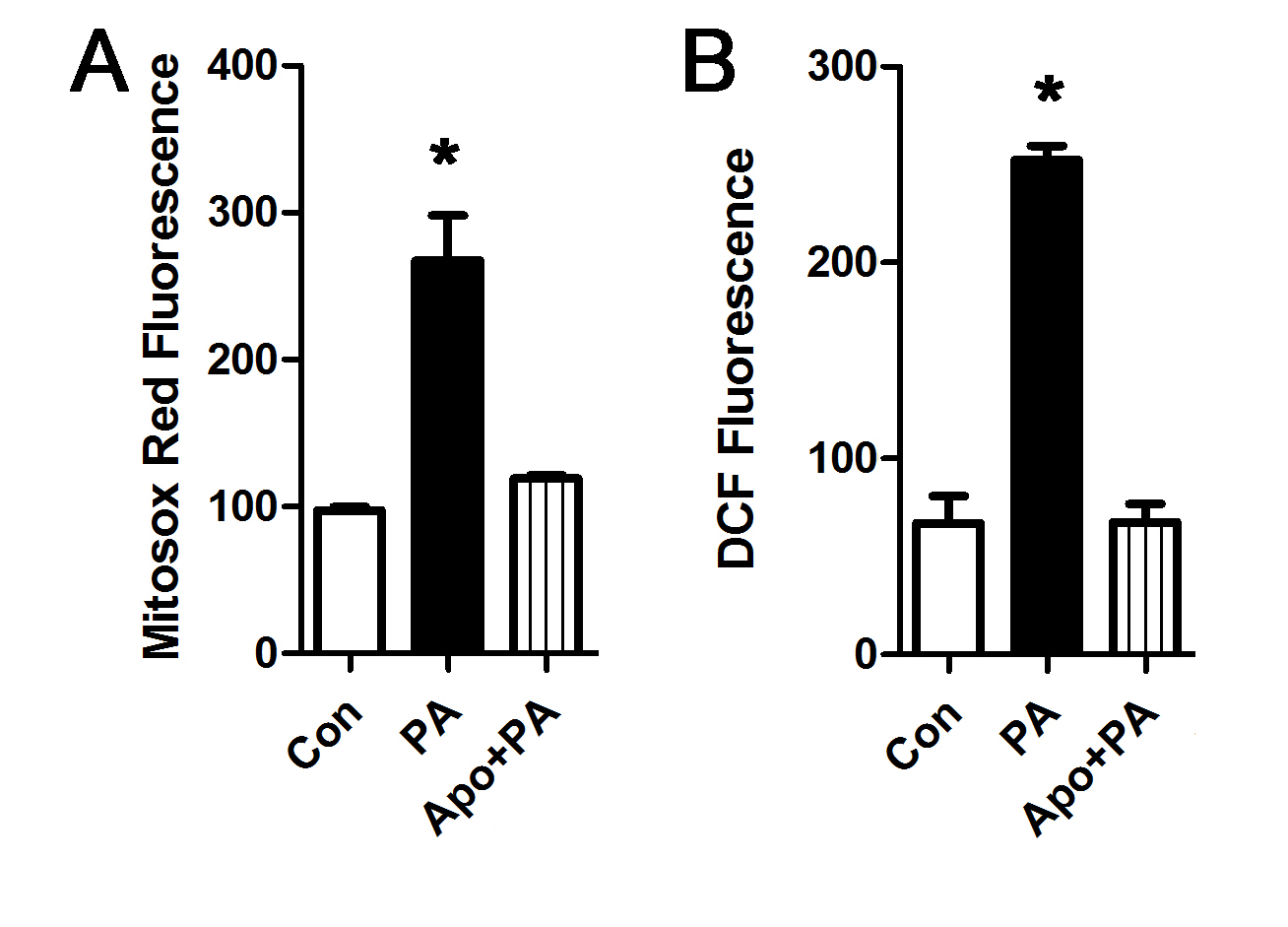

Supplement: S2 File — (DOCX) [file pone.0145750.s002.docx]
