# Supplementary material for: Inhibition of NAPDH Oxidase 2 (NOX2) Prevents Oxidative Stress and Mitochondrial Abnormalities Caused by Saturated Fat in Cardiomyocytes
Source: PLoS One. 2016 Jan 12;11(1):e0145750. doi: 10.1371/journal.pone.0145750 (PMC4710525; doi:10.1371/journal.pone.0145750)

**S3 file: Online Supplement for: Inhibition of NAPDH oxidase 2 (NOX2) prevents oxidative stress and mitochondrial abnormalities caused by saturated fat in cardiomyocytes**

# Supplemental Figure 3. PA activates PKC and PA-induced ROS production is inhibited by blocking NOX2 or PKC in H9c2 cells

A. Representative experiment done in triplicate, height is DCF fluorescence minus background, in H9c2 cells, mean + SEM.

B. Parallel experiment using mitosox red readout.

For all panels, means are significantly different by ANOVA, *= sig different from control by post-hoc test. PA= palmitate 200 μM, apo = apocynin 200 μM, Ln = L-NAME 10 μM, Go = Go6983 5 μM, LY = LY333531 50 nM


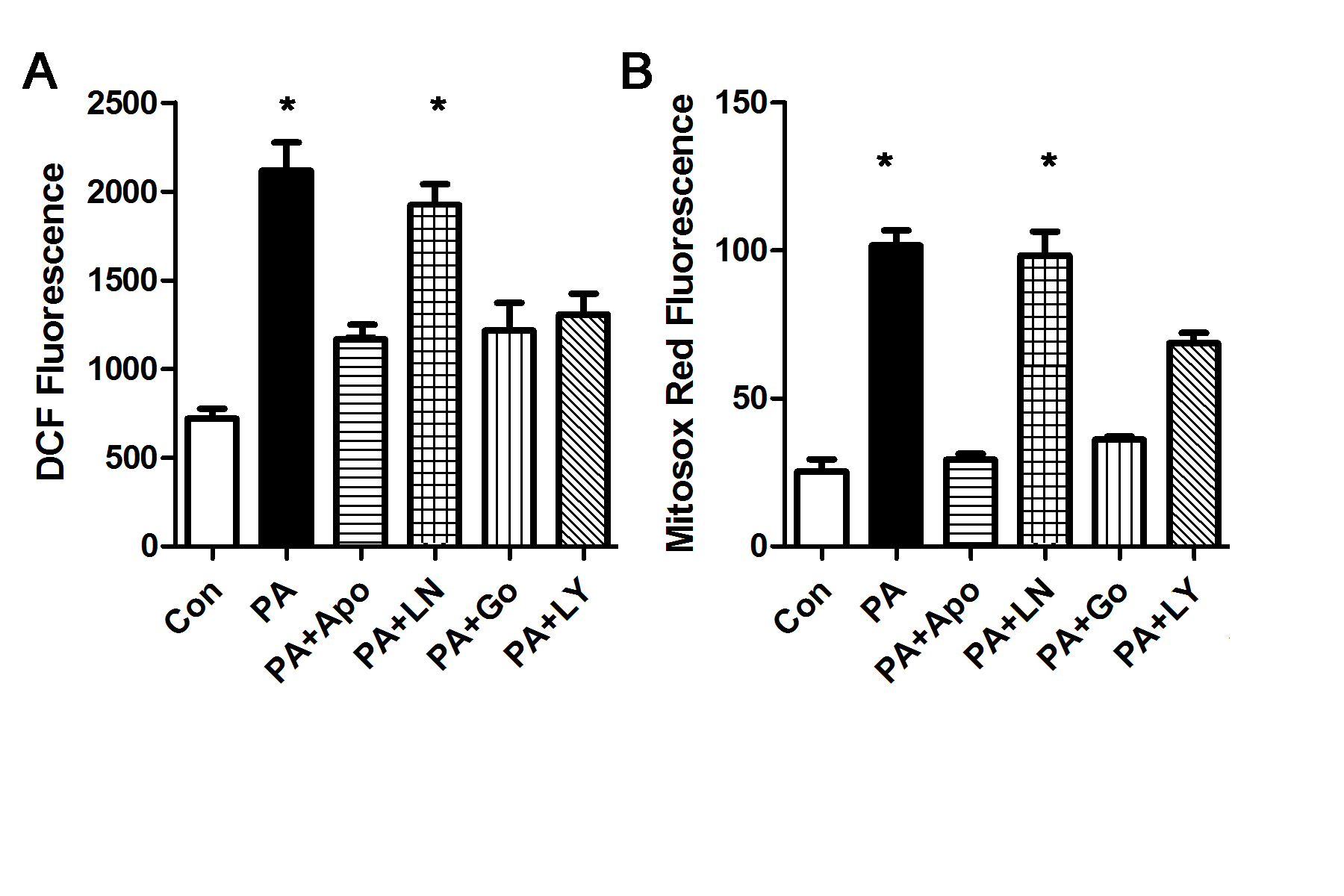

Supplement: S3 File — (DOCX) [file pone.0145750.s003.docx]
