# Supplementary material for: Inhibition of NAPDH Oxidase 2 (NOX2) Prevents Oxidative Stress and Mitochondrial Abnormalities Caused by Saturated Fat in Cardiomyocytes
Source: PLoS One. 2016 Jan 12;11(1):e0145750. doi: 10.1371/journal.pone.0145750 (PMC4710525; doi:10.1371/journal.pone.0145750)
Supplement: S4 File — (DOCX) [file pone.0145750.s004.docx]

**S4 file: Online Supplement for: Inhibition of NAPDH oxidase 2 (NOX2) prevents oxidative stress and mitochondrial abnormalities caused by saturated fat in cardiomyocytes**

**Supplemental** **Figure 4: Mitotempo improves PMA-induced ROS in cardiomyocytes**

A. Representative experiment done with cardiomyocytes in triplicate, height is DCF fluorescence minus background, mean + SEM.

B. PMA experiment using mitosox red readout.

C. PMA experiment using TMRM signal. NOX2 inhibitor and mitotempo reduce depolarization.

D. PMA experiment using Rhod2 signal; NOX2 inhibitor and mitotempo reduce calcium overload.

For all panels, means are significantly different by ANOVA, *= sig different from control by post-hoc test, PMA 100 nM, Apo= apocynin 200 μM, MT= mito-TEMPO, 20 μM.

**
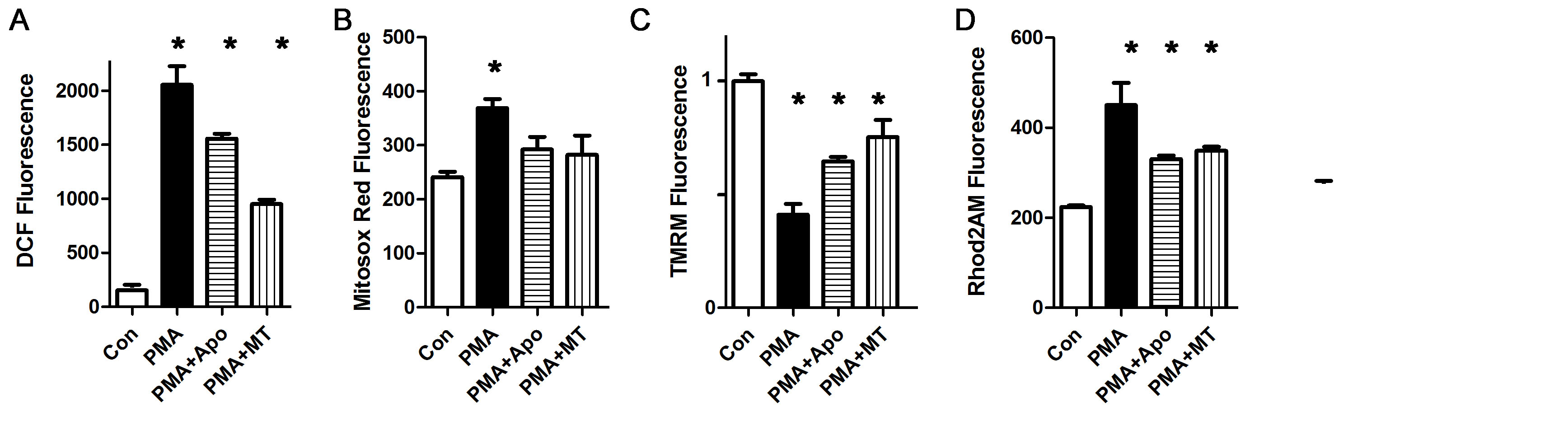
**
